# Anti-Aging Effect of Agar Oligosaccharide on Male *Drosophila melanogaster* and Its Preliminary Mechanism

**DOI:** 10.3390/md17110632

**Published:** 2019-11-06

**Authors:** Chao Ma, Kun Yang, Yifan Wang, Xianjun Dai

**Affiliations:** College of Life Sciences, China Jiliang University, Hangzhou 310018, Zhejiang, China

**Keywords:** agar oligosaccharide, male *Drosophila melanogaster*, anti-aging, antioxidation, gut microbiota, intestinal immunity

## Abstract

Agar oligosaccharide (AOS) is a marine prebiotic with apparent anti-inflammatory, antioxidant and anti-tumor effects. During this study, different doses of AOS are added to a basal diet to evaluate its effects on the lifespan, motor vigor and reproduction of male *Drosophila melanogaster*. Additionally, the activities of Cu,Zn-superoxide dismutase (Cu,Zn-SOD) and catalase (CAT) and the malondialdehyde (MDA) content in male *Drosophila* are examined on the 10th, 25th and 40th days. The fly midguts are removed on the 10th and 40th days for analyses of the intestinal microbial community by 16S rDNA sequencing and the expression level of intestinal immunity genes by quantitative real-time PCR (RT-PCR). The results show that AOS significantly prolonged the average and maximum lifespan and increased the antioxidant capacity of male *Drosophila*. Additionally, AOS significantly regulated the structure of the intestinal flora of "old" flies (40 days) and upregulated the expression of immune deficiency (IMD) genes to improve the intestinal immunity, which could be beneficial for delaying aging in old flies. The above-described results provide a theoretical basis for the application of AOS, a type of marine oligosaccharide, as a nutritional supplement or immunomodulator.

## 1. Introduction

Over the past few decades, seaweed has been considered a promising organism that provides novel bioactive substances and compounds essential for human nutrition [1,2]. Agarose, a marine polysaccharide, is constructed from complex saccharide molecules extracted from certain species of red algae [3]. Agarose has long been used in a variety of laboratory and industrial applications (food, medicine and cosmetics), but the complex structure of agarose polysaccharides makes them resistant to degradation by endogenous enzymes in the human gut [4]. Agar oligosaccharide (AOS) is the product of degradation of agarose, which can be classified as agarose oligosaccharide or new agarose oligosaccharide depending on the reducing end [5]. Compared with polysaccharides, AOS shows better water solubility, easier absorption and other improved physical properties and exhibits biological activities, such as antioxidant activity, anti-tumor, anti-virus and immune activation [6,7,8]. Notably, its effects on senescence have not been investigated yet.

Aging is a normal process in various tissues and organs, and the biological functions of the body degenerate with age. Additionally, the effects of aging are reflected in various behavioral aspects, such as lifespan, exercise vitality, and reproductive ability [9,10], and are related to free radicals, which are a product of oxidation [11]. Under normal conditions, oxidation and antioxidant activity are maintained at a steady state in the body, but increasing age is associated with the metabolization of a higher amount of reactive oxygen species (ROS) into free radicals, which cause oxidative damage to cells [12]. Li et al. reported that the body can resist the effects of aging through a series of enzyme systems, such as superoxide dismutase (SOD), catalase (CAT), and glutathione peroxidase (GPx), which can improve antioxidant activity [13].

As a model organism, *Drosophila* has the advantages of rapid reproduction, a short growth cycle, easy maintenance and operation, and defense systems and aging genes that are similar to those found in humans [14]. *Drosophila* has been established as an emerging and valuable model for research on human-related diseases and the aging mechanism over the past few decades, but only recently has been used for intervention studies on nutrition [15]. Studies have revealed significant gender differences in the aging mechanism of *Drosophila* and, based on the differences in hormones and reproduction between female and male *Drosophila*, male *Drosophila* constitute a more suitable biological model for anti-aging studies [16,17].

There is a complex relationship between aging and the intestinal microflora of the body, and the intestinal microflora and barrier function of *Drosophila* before senescence are significantly different from those after senescence [18]. “Old” flies tend to exhibit a disordered composition of intestinal microbes and an impaired intestinal barrier function [19], but prebiotics can selectively enhance the beneficial components of the intestinal microflora of *Drosophila* [20,21].

## 2. Results

### 2.1. Effect of AOS on the Lifespan of Drosophila

**Figure 1:**
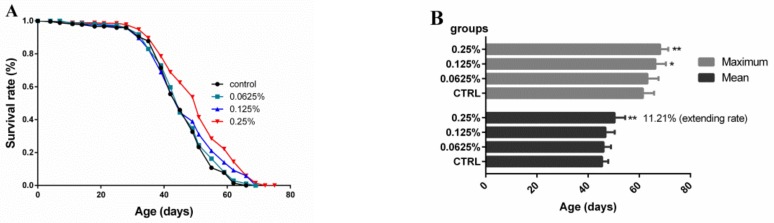
Analysis of the lifespan of *Drosophila* fed diets containing various AOS doses. The lifespan was analyzed on different days. Lifespan (**A**) and mean and maximal lifespan (**B**) of *Drosophila* in the various oligosaccharide test groups or the standard diet control group. The results are presented as the means ± SEMs (*n* = 9). Each group included nine biological replicates; * *p* < 0.05 and ** *p* < 0.01 indicate significant differences.

The overall survival rate of each group of fruit flies throughout the experiment is shown in Figure 1A. The results showed that the survival rate of *Drosophila* increased with increases in the oligosaccharide dose. We then analyzed the average lifespan, maximum lifespan and average life extension rate of each group (Figure 1B). Compared with the control group, the average lifespan of *Drosophila* in the high-dose group was prolonged significantly (50.37 ± 1.38 vs. 45.50 ± 0.80 days), and the average life extension rate was 11.21% (*p* < 0.01). However, no significant difference in the average lifespan was found between the low- and medium-dose groups (46.06 ± 0.96 and 46.83 ± 1.20 vs. 45.50 ± 0.80 days, respectively). Additionally, the maximum lifespans of the middle-dose and high-dose groups were increased significantly (66.33 ± 1.36 and 68.22 ± 1.05 vs. 61.44 ± 1.45 days, *p* < 0.05, respectively) compared with that of the control group. According to the above-described results, the oligosaccharide dose exerted a significant effect on the lifespan, and the inclusion of an AOS amount in the diet equal to at least the medium dose resulted in a significant increase in the lifespan.

### 2.2. AOS Improved the Vitality of Juvenile Fruit Flies

**Figure 2:**
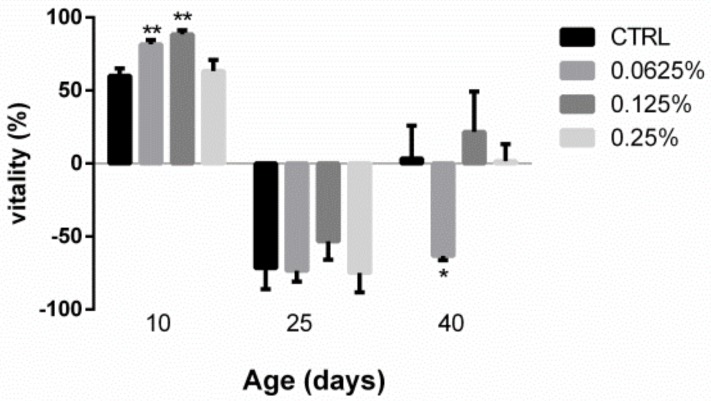
Vitality of *Drosophila* fed diets containing different AOS contents. The vitality was assessed on different days. The results are presented as the means ± SEMs (*n* = 3). Each group included three biological replicates; * *p* < 0.05 and ** *p* < 0.01 indicate significant differences.

Shown in Figure 2, the activity of *Drosophila* in the low-dose and middle-dose groups was 81.67 ± 1.67% and 88.33 ± 1.67%, respectively, on the 10th day, and these values indicated significant increases of 36.11% (*p* < 0.01) and 8.16% (*p* < 0.01), respectively, compared with that found for the control group. Regarding the 25th and 40th days, no significant differences in vitality were found between the treated flies and the control group, with the exception of the low-dose group on the 40th day.

### 2.3. AOS Reduced the Reproductive Capacity of Drosophila

**Figure 3:**
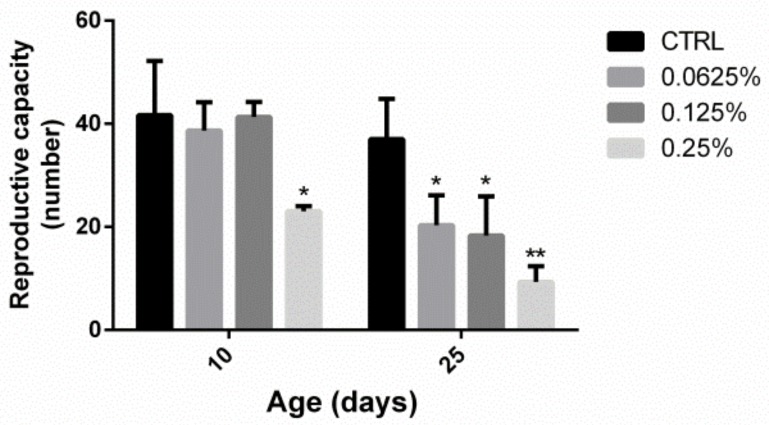
Reproductive capacity of *Drosophila* fed diets containing different AOS contents. The reproductive capacity was measured on different days. The results are presented as the means ± SEMs (*n* = 3). Each group included three biological replicates; * *p* < 0.05 and ** *p* < 0.01 indicate significant differences.

Shown in Figure 3, the reproductive capacity of *Drosophila* in the high-dose group was lower than that of the control group on the 10th day (23.00 ± 0.57 vs. 41.67 ± 6.06, *p* < 0.05), but no significant differences were found between the medium- and low-dose groups (38.67 ± 3.18 and 41.33 ± 1.667, respectively) and the control group. Concerning the 25th day, the fertility of the low-, medium- and high-dose groups was reduced significantly compared with that of the control group (20.33 ± 3.33, 18.33 ± 4.37 and 9.333 ± 1.76, respectively, vs. 37.00 ± 4.51, *p* < 0.05). Furthermore, the reproductive ability decreased with increases in the oligosaccharide dose.

### 2.4. AOS Improved the Antioxidant Capacity of Drosophila

**Figure 4:**
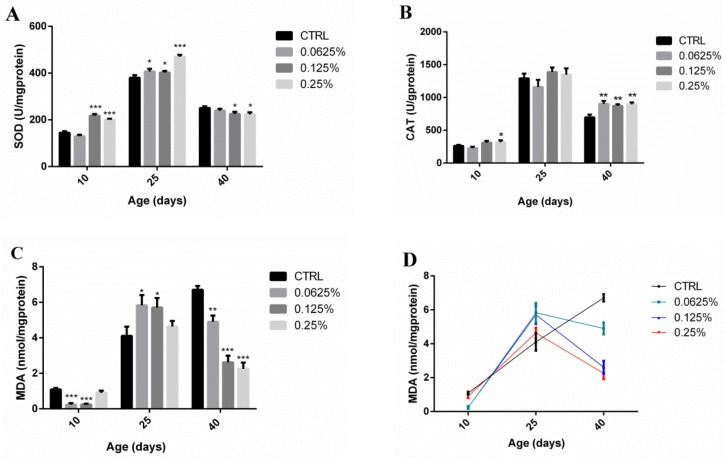
Antioxidant capacity of *Drosophila* fed diets containing different AOS contents. The antioxidant capacity was measured on different days. The activities of Cu,Zn-SOD (**A**) and CAT (**B**) and MDA contents (**C**,**D**) were measured in whole tissue of *Drosophila*. The results are presented as the means ± SEMs (*n* = 3). Each group included three biological replicates; * *p* < 0.05, ** *p* < 0.01, and *** *p* < 0.001 indicate significant differences.

Regarding the 10th and 25th days, the Cu,Zn-SOD enzyme activity in the test groups was higher than that of the control group (217.5 ± 4.09 and 200.0 ± 2.86 vs. 144.6 ± 3.97 U/mg protein, *p* < 0.05; 406.5 ± 6.88, 402.4 ± 4.01 and 470.4 ± 4.43 vs. 380.2 ± 6.27 U/mg protein, *p* < 0.05) (Figure 4A). Concerning the 10th day, the CAT activity of the high-dose group was increased significantly compared with that of the control group (317.6 ± 16.99 vs. 263.3 ± 7.71 U/mg protein, *p* < 0.05), and on the 40th day, the CAT activities of the low-, medium- and high-dose groups were all higher than that of the control group (902.8 ± 26.00, 872.8 ± 14.44 and 896.4 ± 17.29, respectively, vs. 697.5 ± 23.86 U/mg protein, *p* < 0.01) (Figure 4B). Regarding the 10th day, the malondialdehyde (MDA) contents of the low-dose and medium-dose groups were significantly lower than that of the control group (0.2397 ± 0.047 and 0.2414 ± 0.028, respectively, vs. 1.092 ± 0.048 nmol/mg protein, *p* < 0.001), and on the 40th day, the MDA contents in the low-, medium- and high-dose groups were all lower than those of the control group (4.902 ± 0.20, 2.617 ± 0.22 and 2.254 ± 0.20, respectively, vs. 6.708 ± 0.13 U/mg protein, *p* < 0.01) (Figure 4C). The trend obtained for the MDA content in the experimental groups consisted of an initial increase followed by a decrease, whereas a continuous increase in the MDA content over time was observed in the control group (Figure 4D).

### 2.5. Analysis of the Diversity of the Midgut Microflora in Old Flies by 16S rDNA Sequencing

**Figure 5:**
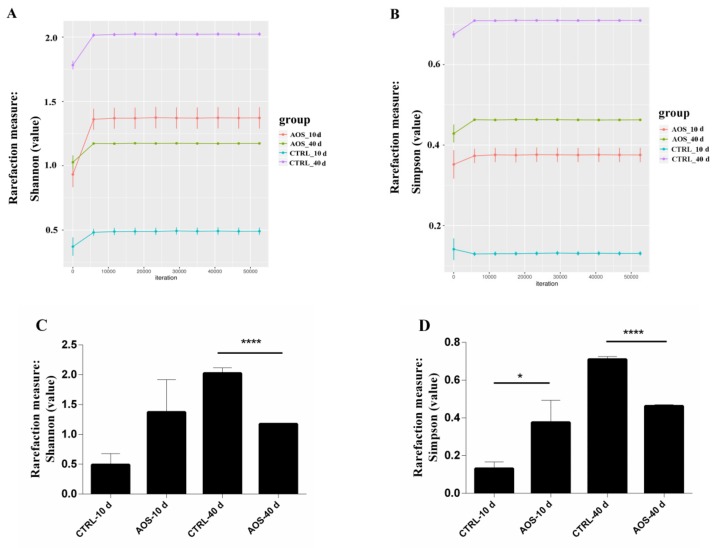
Abundance index of the microbial community in the midgut of the AOS-fed and control groups. The abundance index was measured on different days. The dilution curves include the Shannon index (**A**) and the Simpson index (**B**), and contrast histograms of the dilution index include the Shannon index (**C**) and the Simpson index (**D**) of the alpha diversity of sample species abundance. The results are expressed as the means ± SEMs (*n* = 3). Each group included three biological replicates; * *p* < 0.05 and **** *p* < 0.0001 represent significant differences.

The microbial community diversity was assessed using the Shannon and Simpson indexes, and the obtained dilution curves for the Shannon index (Figure 5A) and Simpson index (Figure 5B) revealed that the microbial diversity in the experimental group was reduced significantly compared with that of the control group on the 40th day. Shown in Figure 5C, the Shannon indexes of the control and experimental groups on the 40th day were 2.02 ± 0.053 and 1.17 ± 0.0022, respectively (*p* < 0.01) and, as shown in Figure 5D, the corresponding Simpson indexes were 0.71 ± 0.0088 and 0.46 ± 0.0041, respectively (*p* < 0.01). Based on these findings, AOS significantly reduced the microbial diversity of the midgut of old flies.

**Figure 6:**
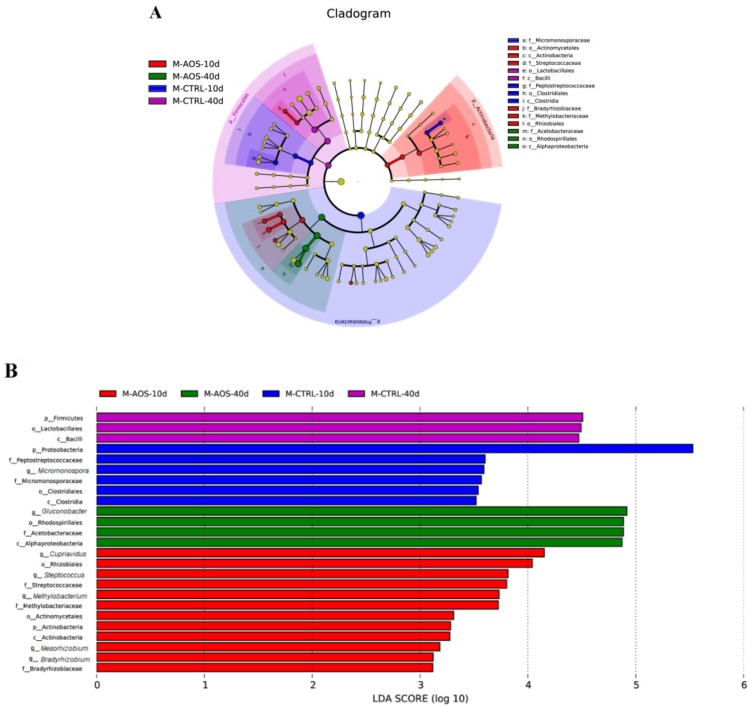
Cladograms of bacterial lineages showing significant differences between the AOS-fed and control groups. The cladogram in (**A**) shows the relative abundance of dominant bacteria in the *Drosophila* intestine that met a significant LDA threshold value of >2 in the AOS-fed and control groups. The bacterial community from the phylum to the genus level is provided from the center to the outside, respectively. Different colors represent different groups and the yellow node represents a group of microbes that do not play important roles in the different groups. An LEfSe analysis of the 16S sequence was used to obtain a taxonomic cladogram and estimate the effective proportional abundance of each component. The lengths represent the effect size of the bacterial lineages (**B**).

A linear discriminant analysis effect size (LEfSe) analysis using a linear discriminant analysis (LDA) score log10 > 3 was applied to assess the specific changes in the bacterial population induced by AOS. According to the LDA score, 25 taxa exhibited a significant difference in abundance between the AOS-fed and control groups. Regarding the 40th day, the relative abundances of *Alphaproteobacteria*, *Rhodospirillales*, *Acetobacteraceae* and *Gluconobacter* (from the class to the genus levels) in the AOS-fed group were increased significantly (LDA value > 4.0) compared with those found in the control group, and those of Firmicutes, Bacilli and *Lactobacillus* (from the phylum to the order levels) were decreased significantly (LDA > 4.0) in the test group compared with the control group (Figure 6A). Concerning the 10th day, the phylum Proteobacteria in the control group showed the largest effect size compared with other phyla (LDA > 5.0) (Figure 6B).

**Figure 7:**
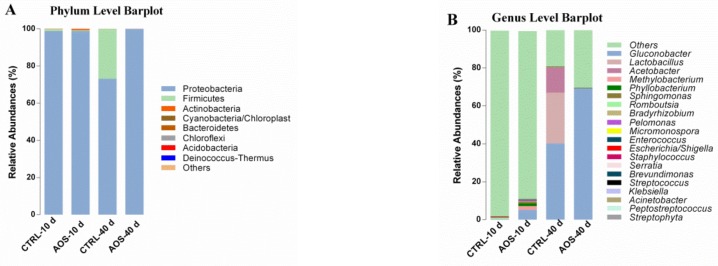
Relative abundance of dominant microbial species at the phylum (**A**) and genus (**B**) levels in the midgut of *Drosophila*. The term “Others” indicates bacteria that were not included in the information database. The data presented for each group in the figure are the average value from three biological replicates.

The phylum-level analysis revealed higher abundances of Proteobacteria, Firmicutes and *Actinobacteria*, and their respective proportions in the various groups were the following: CTRL_10d, 98.78%, 1.00%, and 0.11%; AOS_10d, 98.69%, 0.54%, and 0.57%; CTRL_40d, 67.29%, 32.64%, and 0.067%; AOS_40d, 99.91%, 0.023%, and 0.058%. Regarding the 40th day, the abundances of Proteobacteria and Firmicutes were increased significantly (*p* < 0.01) and significantly decreased (*p* < 0.01), respectively, in the experimental group compared with the control group (Figure 7A). Occurring at the genus level, the dominant species with higher abundances were *Gluconobacter*, *Lactobacillus*, *Acetobacter* and *Methylobacterium*. Concerning the 40th day, *Gluconobacter* was the most abundant genus in the experimental group, with an average ratio of 69.18%, and this value was 28.99% higher than that found for *Gluconobacter* in the control group (*p* < 0.001). The abundances of *Lactobacillus* and *Acetobacter* were notably lower in the experimental group compared with the control group (*p* < 0.01) (Figure 7B).

### 2.6. Immune Defense-Related Gene Expression in Senile Flies Fed Diets Containing AOS

**Figure 8:**
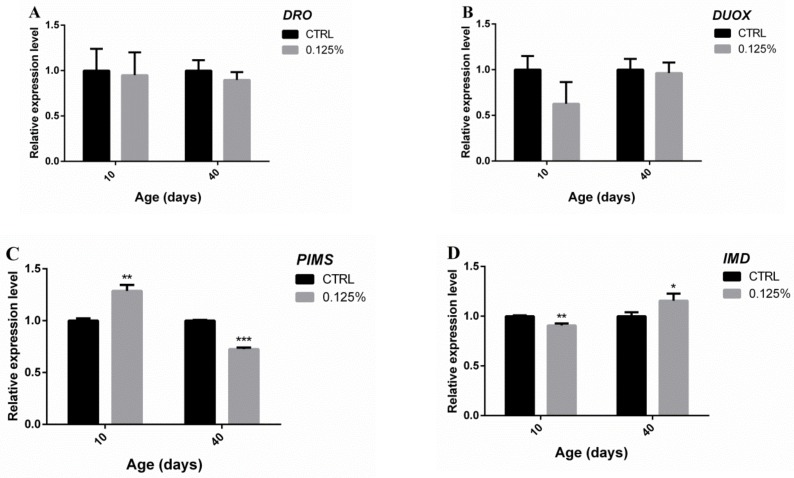
Fluorescence quantitative PCR detection of the expression of *DRO* (**A**), *DUOX* (**B**), *PIMS* (**C**), and *IMD* (**D**) in the flies in the experimental and control groups on the 10th and 40th days. The results are presented as the means ± SEMs (*n* = 3). Each group included three biological replicates; * *p* < 0.05 and ** *p* < 0.01 indicate significant differences.

Shown in Figure 8, no significant difference in the expression levels of drosocin (*DRO*) or *DUOX* was found between the experimental and control groups on the 10th day. To contrast, compared with the control group, the expression of *PIMS* and *IMD* was upregulated (1.286 ± 0.033 vs. 1.000 ± 0.012, *p* < 0.01) and downregulated in the experimental group (0.9071 ± 0.011 vs. 1.000 ± 0.0047, *p* < 0.01). Regarding the 40th day, no significant difference in the expression of *DRO* or *DUOX* was found between the experimental and control groups. Interestingly, compared with the control group, the expression of *PIMS* and *IMD* was decreased significantly (0.7257 ± 0.0083 vs. 1.000 ± 0.0038, *p* < 0.01) and significantly upregulated (1.155 ± 0.041 vs. 1.000 ± 0.023, *p* < 0.05) in the experimental group.

## 3. Discussion

Marine oligosaccharides have attracted widespread attention due to their various biological activities. Previous studies have demonstrated some of the biological activities of chitooligosaccharides (COSs), alginate oligosaccharides (AlgOs) and carrageenan oligosaccharides (CAOs), such as the anti-tumor efficacy of COSs and their derivatives [22,23] and the anti-tumor and immunomodulatory functions of AlgOs and CAOs [24,25,26]. However, few in vivo studies have investigated the anti-aging, antioxidant and immunomodulatory effects of AOS or the associated mechanisms of action. Accumulating evidence from recent studies indicates that oligosaccharides, such as marine prebiotics, could stabilize the intestinal environment of the host by promoting the growth of beneficial bacteria, and a close relationship among gut microbiota, immunity and aging has been detected [27,28].

Our study found that AOS, particularly at a high dose, can improve significantly the average life expectancy and maximal lifespan of males. The motor vigor of young flies fed in the middle-dose and low-dose groups also was improved significantly, but AOS exerted no significant effect on the vigor of "middle-aged" flies. Additionally, an analysis of the reproductive capacity of each group showed that the females in the "older" group lost their reproductive ability, and the reproductive ability of the tested groups at the young and adult stages was reduced significantly. Additionally, the effect of AOS on the reproductive ability of *Drosophila* increased with increasing age. According to previous studies, lifespan is the most intuitive manifestation of the degree of aging [9], and exercise vitality is negatively correlated with aging [10]. Although the effects of aging on the regulatory mechanism of the reproductive system remain unclear [29], some studies have shown that reproduction, which involves physical exertion, exhibits a trade-off with aging, and many species can increase their lifespan by reducing their fertility [30,31]. These findings indicate that a certain amount of AOS effectively can delay senescence in male fruit flies and that these effects might be partly related to a decrease in reproductive ability.

To further explore the anti-aging mechanism of AOS, we analyzed the antioxidant capacity of *Drosophila*. According to a previous study, SOD and CAT are involved in the antioxidant defense network in humans, and the MDA content might reflect the degree of lipid peroxidation in vivo [32]. The simultaneous overexpression of the genes encoding SOD and CAT, for instance, could extend the lifespan of fruit flies [33]. The present study showed that AOS significantly increased the SOD activity in *Drosophila* on the 10th and 25th days, whereas an increase in CAT activity and a decrease in the MDA content were observed mainly in young and, in particular, old *Drosophila*. Reported by Zou, the effects of prebiotics on the antioxidant capacity of old flies are more pronounced than those observed in young flies [9].

Similar to that of mammals, the *Drosophila* gut contains various colonizing microorganisms, and the gut microbiota simultaneously can affect a variety of aging-related biological processes, including inflammation, oxidative stress, and metabolic regulation by regulating the immune system and providing nutrition [34]. The evaluation of the microbial differences between fruit flies revealed that the microbial content of *Drosophila*, independent of species uniformity, shows similarity between species that are fed the same diet [35]. Our microbiological analysis of the *Drosophila* gut showed that supplementation of the standard diet with AOS could change the dominant microorganism in the old *Drosophila* intestine. Concerning the 40th day, the intestinal microbial diversity of the experimental group was reduced significantly compared with that found for the control group and, at the phylum level, the abundances of Proteobacteria and Firmicutes were increased significantly (*p* < 0.01) and significantly decreased (*p* < 0.01) in the experimental group compared with the control group. Notably, Ley et al. reported that the abundance of Firmicutes in the intestinal microbiota of obese individuals was higher than that of normal individuals [36]. Similarly, Zhao et al. reported that the abundance of Firmicutes in the intestine of older pigs (6 months) was 10 times higher than that found in piglets (1 month). [37]. Moreover, the most dominant species of Proteobacteria and Firmicutes belonged to *Acetobacter* and *Lactobacillus spp*., respectively, and *Lactobacillus* species are usually more abundant in old flies than in young flies [38]. During this study, the number of dominant genera in the *Drosophila* intestinal tract was reduced from four to one during aging. Additionally, the number of *Lactobacillus* species in the intestine of old flies was reduced significantly in the experimental group during aging, and *Gluconobacter* was the only dominant genus in the intestines of old flies. According to a previous study, the microflora of *Drosophila* fed diets containing complex polysaccharides, such as cornmeal or soy flour, is often composed of *Lactobacillus* species (*Lactobacillus* of the phylum Firmicutes) at a high abundance. However, the microbiome group in *Drosophila* fed a sugar-rich diet is usually dominated by *Acetobacter* (α-Proteobacteria), particularly *Acetobacter* and *Gluconobacter* [39]. Ryu et al. reported that nuclear factor kappa B (NF-κB) can activate antimicrobial peptides (AMPs) that regulate the composition of intestinal symbiotic bacteria, particularly *Gluconobacter* [40]. Consequently, the old AOS-fed flies exhibited a higher abundance of *Gluconobacter*, which might be related to the oligosaccharide content in the diet and its immune regulation. According to the above-described studies and the results obtained in our experiment, old flies fed AOS exhibit a healthy or younger intestinal microbiota.

Routy et al. reported that intestinal microbes are related closely to intestinal immunity [41]. Since immunization involves a systemic response, we used whole flies for the quantitative RT-PCR analysis, and we tested four genes that play important roles in gut immunity: *DRO*, *DUOX*, *IMD*, and *PIMS*. A previous study showed that in infected *Drosophila*, high expression of *DRO* could improve the integrity of the intestinal barrier, delay senescence of the intestines and organs, and ultimately extend the lifespan [42]. *DUOX*, a key gene in intestinal immunity, is usually activated after intestinal infection, which could lead to “promoted catabolism” signaling, initiate intestinal cell metabolism and reprogrammed lipid catabolism [43]. *IMD* is another gene related to the intestinal immunity of *Drosophila* that initiates the NF-κB signaling pathway [44,45,46]. *PIMS* is an inhibitor of the interaction of the peptidoglycan recognition protein (PGRP-LC), which is an activating receptor for the *IMD* cascade and a negative regulator of *IMD* and is usually upregulated in the presence of commensal bacteria to suppress an immune response [47]. Since the intestinal tract of old *Drosophila* usually reflects the effects of aging and an impaired intestinal barrier, the intestinal immune response likely plays an important role in delaying senescence in *Drosophila* [18]. During our experiment, the expression of *DRO* and *DUOX* showed no significant difference between the experimental and control groups, and the expression of *PIMS* and *IMD* was decreased significantly (*p* < 0.01) and significantly upregulated (*p* < 0.05), respectively, in the experimental compared with the control group. The upregulation of *IMD* in senile flies activated the NF-κB signaling pathway, which was consistent with the high abundance of *Gluconobacter* in the gut of senile flies. Based on the observed decreases in the diversity of intestinal microflora and the abundance of Firmicutes, we found that a certain amount of AOS in the diet effectively could delay aging in male flies. The results also demonstrated that AOS could improve the intestinal immunity of senile flies, as demonstrated by the observed changes in intestinal symbiotic bacteria, but whether the intestinal immunity is improved by these changes in the intestinal flora remains to be addressed in a future study.

## 4. Materials and Methods

### 4.1. Animals, Diets and Sample Preparation

The Canton-S line of *Drosophila melanogaster* was obtained from the *Drosophila* Stock Center at Shanghai Academy of Life Sciences, Chinese Academy of Sciences. The flies were raised at 24 ± 1 °C under 55% relative humidity with a 12-h light/12-h dark cycle. Pharmaceutical-grade AOS (≥95%) was purchased from Qingdao Bozhi Huili Biotechnology Co., Ltd., China (Qingdao, China). The AOS was obtained through the acid hydrolysis of agar that was mainly composed of agarobiose (A2), agarotetraose (A4) and agarohexaose (A6), as determined by HPLC [48]. During this study, the control fruit flies were housed in standard medium composed of corn-yeast-agar. Preliminary in vitro experiments were performed to determine the optimal concentration for in vitro administration, which was determined according to the following formula: experimental in vitro drug concentration (μg/mL) = 50 × D/5000 ÷ 50% × 10^3^ (D: clinical dose mg·kg day). The optimal dose of AOS for the *Drosophila* in our experiment was calculated to equal 0.125%. Three experimental groups were established to assess low, medium and high doses of AOS, and 0.0625%, 0.125%, and 0.25% AOS was added to the standard diet fed to these groups, respectively. MDA, Cu,Zn-SOD and CAT kits and protein standards were purchased from Nanjing Jiancheng Bioengineering Co., Ltd. (Nanjing, China).

### 4.2. Lifespan Assay [49,50]

Newly emerged *Drosophila* in a standard culture medium were divided randomly into four groups under slight anesthesia with carbon dioxide gas. Thirty flies were included in each tube, and each group consisted of 10 tubes. The food was replaced every 3–4 days, and the number of fruit fly deaths was recorded until all the flies were dead. The average lifespan, maximal lifespan, and average life extension rate were obtained for each group of fruit flies.

### 4.3. Climbing Ability Assay [51]

During the experiment, a negative geodetic was used to study the effects of different doses of AOS on the motor vigor of *Drosophila*. Newly emerged fruit flies were randomly divided into four groups according to the above assay; each group comprised three tubes, and each tube contained 20 flies. The tubes were tapped until all the flies reached the bottom of the tube. Ten seconds later, the number of fruit flies at the top and bottom was recorded. The number of fruit flies on the top was subtracted from the number of flies on the bottom, and the percentage was calculated. This analysis of *Drosophila* exercise activity was performed on the 10th day, 25th day, and 40th day of culture.

### 4.4. Reproduction Assay [9]

Newly emerged fruit flies were divided randomly into four groups according to the above experiment. Each group consisted of 10 tubes of males and females, and each tube contained three flies. Male and female *Drosophila* were mixed in five tubes for free mating on the 10th and 25th days of culture. Following 5 days of mixed culture, the parental *Drosophila* were removed, and the culture was continued. The total number of fruit flies in each tube was recorded for 7 days starting on the day on which the first *Drosophila* emerged.

### 4.5. Antioxidation Assay

*Drosophila* were collected after 10, 25, and 40 days in culture, fasted for 2 h and weighed, and the average body weight was recorded. The fruit flies were mixed with physiological saline, in an ice bath, at the fruit fly weight:physiological saline ratio of 1:9. The mixture then was homogenized with a glass homogenizer and centrifuged at 4 °C and 560 g/min for 15 min. The supernatant was collected, and the MDA content of the mixture was measured. The tissue homogenate was diluted to 5% for measurements of the SOD and CAT activities. The detection of the MDA content and the SOD and CAT activities was performed according to the manufacturer’s instructions.

### 4.6. 16S rDNA Sequencing

The optimal oligosaccharide dose was determined according to the above experimental results, and the newly emerged fruit flies were randomly divided into two groups (test group with 0.25% AOS and standard diet control group). “Young” and “old” *Drosophila* were collected on the 10th and 40th days, respectively. The bodies of the fruit flies were sterilized with 75% ethanol and bleached with 10% Tween phosphate-buffered saline (TPBS) for 1 min. The fruit flies were dissected on ice, and the midgut was removed. The midgut samples were sent to MicroAnaly Gene Technologies Co., Ltd. (Anhui, China), for analysis of the microbial community. Briefly, the midgut DNA was extracted using the OMEGA stool DNA Kit (Omega Bio-tek, Norcross, GA, USA). PCR amplification was performed by targeting the 16S rRNA gene sequence (region V6–V8), and libraries were prepared according to the guidelines provided by Illumina and sequenced using the Microbiome Helper workflow. Open-reference operational taxonomic unit (OTU) selection was performed based on 97% identity using Usearch_v11 (version 2.7.1, Sonoma, CA, USA), and the reads were clustered against the Greengenes database. The final OTU table was normalized to each sample using DESeq2.

### 4.7. Quantitative RT-PCR Analysis

Using the same method, the other sections of the *Drosophila* midgut were collected on the 10th and 40th days, triturated with TRIzol reagent in liquid nitrogen, and centrifuged to obtain total RNA, which was reverse transcribed to synthesize cDNA. Using the ribosomal protein 49 (rp49) expression level as an internal reference [52], qPCR then was performed to detect the mRNA expression levels of genes related to the gut immune system. The primer sequences and annealing temperatures are shown in Table 1 (primers were designed and synthesized by Wcgene Biotech, Shanghai, China).

### 4.8. Statistical Analysis

The mRNA expression levels of *DRO*, *DUOX*, *PIMS* and *IMD* were determined using Rp49 as an internal reference, and each relative expression level of mRNA was calculated using the 2^−ΔΔ*C*t^ method. No less than three biological replicates were included for each experimental group, and the intestinal microbes collected data were combined in this study and analyzed using GraphPad Prism 6 (Version No.6.01, GraphPad Software, La Jolla, CA, USA). Statistical significance was set to *p* < 0.05.

## 5. Conclusions

Analyses of aging, antioxidant and immunomodulatory effects and intestinal microbes were combined in this study to explore the anti-aging effect of AOS as a marine prebiotic for *Drosophila*. A certain amount of AOS in the male fly diet could delay aging, as demonstrated by the extended lifespan and improvement in exercise vitality. Based on our findings, these effects might be related to a decrease in reproductive ability, an increased antioxidant capacity, amelioration of the intestinal microbiota and the regulation of intestinal immunity. However, further research is needed to obtain a better understanding of the anti-aging mechanism of AOS, including the associated cellular pathways and the transcriptome/metabolome of gut microorganisms or the gut itself.

## Figures and Tables

**Table 1:** Real-time PCR primers of immune defense-related genes in *Drosophila*.

Gene Name	Sequence 5′-3′	Annealing Temp
*Duox*	F:CAGTTTCGAACGAACTCTTGG R:CATGGTTTTCCAATTATGCAGATTC	54 °C
*IMD*	F:AAAACGGTCAGCATTGTGGC R:GTCCTTCCGACATGCCAAGA	52 °C
*Drosocin*	F:GCACAATGAAGTTCACCATCGT R:CCACACCCATGGCAAAAAC	56 °C
*PIMS*	F:ATCGTTTTCCTGCTGCTTGC R:ATTACTTGCAGTTGCCCGGA	59 °C
Rp49	F:AGGGTATCGACAACAGAGTG R:CACCAGGAACTTCTTGAATC	52 °C
